# Supplementary material for: Multi-scale quantification and modeling of aged nanostructured silicon-based composite anodes
Source: Commun Chem. 2020 Oct 16;3:141. doi: 10.1038/s42004-020-00386-x (PMC9814897; doi:10.1038/s42004-020-00386-x)
Supplement: Supplementary file 1 — Supplementary Information [file 42004_2020_386_MOESM1_ESM.pdf]

Supplementary Information

Multi-scale quantification and modeling of aged nanostructured silicon-based composite anodes

Thomas Vorauer[1], Praveen Kumar[2], Christopher L. Berhaut[3], Fereshteh F. Chamasemani[1], Pierre-Henri Jouneau[2], David Aradilla[3], Samuel Tardif[2], Stephanie Pouget[2], Bernd Fuchsbichler[4], Lukas Helfen[5,6], Selcuk Atalay[7], Widanalage D. Widanage[7], Stefan Koller[4], Sandrine Lyonnard[3], Roland Brunner[1*]

[1] Materials Center Leoben Forschung GmbH, A8700 Leoben, Austria

[2] Univ. Grenoble Alpes, CEA, IRIG-MEM, Grenoble 38000, France

[3] Univ. Grenoble Alpes, CEA, CNRS, IRIG, SyMMES, Grenoble 38000, France

[4] Varta Micro Innovation GmbH, A8010 Graz, Austria

[5] Institute for Photon Science and Synchrotron Radiation, Karlsruhe Institute of Technology, D-76131 Karlsruhe, Germany

[6] Institut Laue–Langevin, CS 20156, 38042 Grenoble Cedex 9, France

[7] WMG, University of Warwick, Coventry, CV4 7AL, UK

SUPPLEMENTARY FIGURES

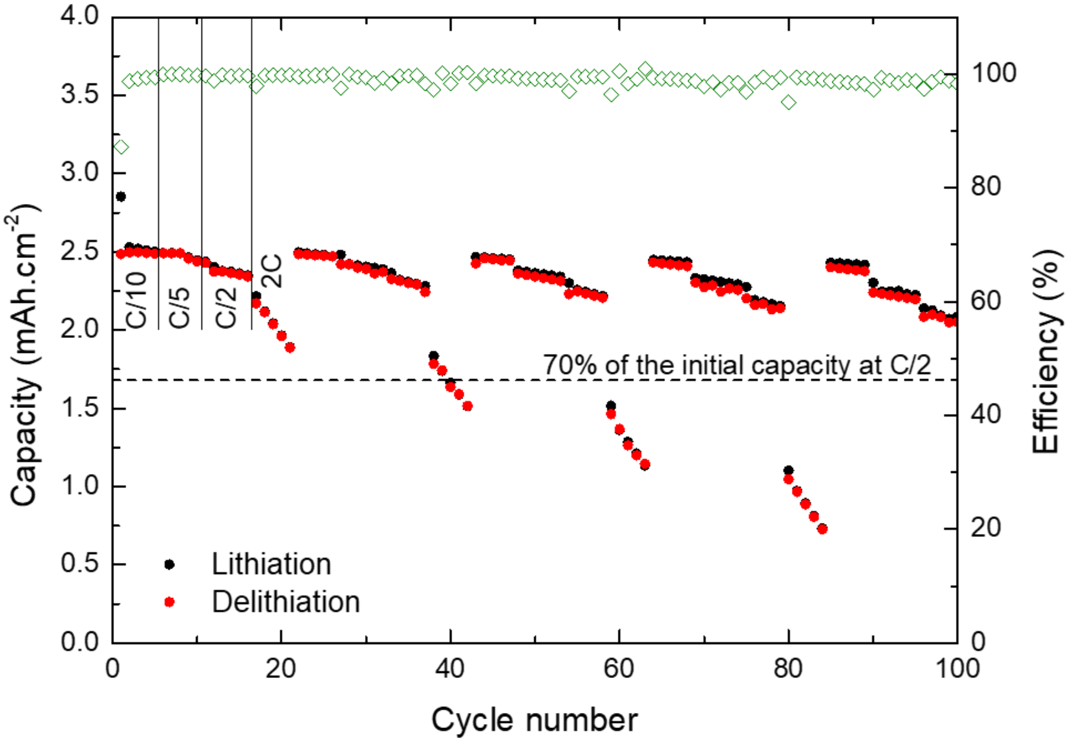

Supplementary Figure 1| Electrochemical characterization. Capacity retention of a-Si/$FeSi_2$/graphite//Li half-cell at different C-rates (current rates). Red and green dots represent the lithiation and delithiation process, and efficiency (green diamonds), respectively. This plot shows the high capacity retention at C-rates lower and equal to C/2 of the a-Si/c-$FeSi_2$/graphite electrode. After 100 cycles, the cell capacity has yet to pass below 70 % of the initial capacity measured at C/2. However, when the cell is cycled at 2C the capacity drops fast due to polarization of the electrode. According to the results in this figure, it is estimated that the cell capacity at C/2 should reach 70 % of its initial value after completing at least 200 cycles following the same procedure. This is in agreement with the results obtained for a 30 mAh full-cell prepared with a 1/1/1 Nickel-Manganese-Cobalt-Oxide (NMC) electrode as cathode (see

Supplementary Note 1). The discharge capacity fading of the NMC//a-Si/$FeSi_2$/graphite pouch-cell is close to 30 % after 321 cycles at C/2.

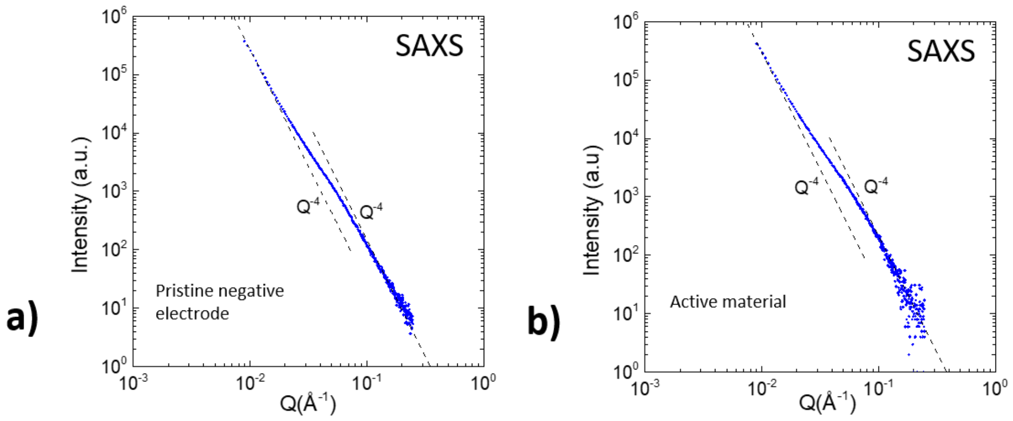

Supplementary Figure 2| Lab-SAXS measurement. Scattering intensity profiles measured by lab-SAXS of the a-Si/c-$FeSi_2$/graphite powder (**a**), and of the pristine negative electrode (**b**) as a function of the scattering vector Q. The 1D SAXS profiles are very similar in both the powder and the pristine electrode. Two Porod's behavior (intensity decay following a Q^{-4} power law) are observed at low and high Q, indicating the presence of neat large-scale (>60 nm) and smaller-scale (~10 nm) interfaces in the materials. The experiments were performed on the SAXS camera at CEA-IRIG (Cu λ = 1.54 Å).

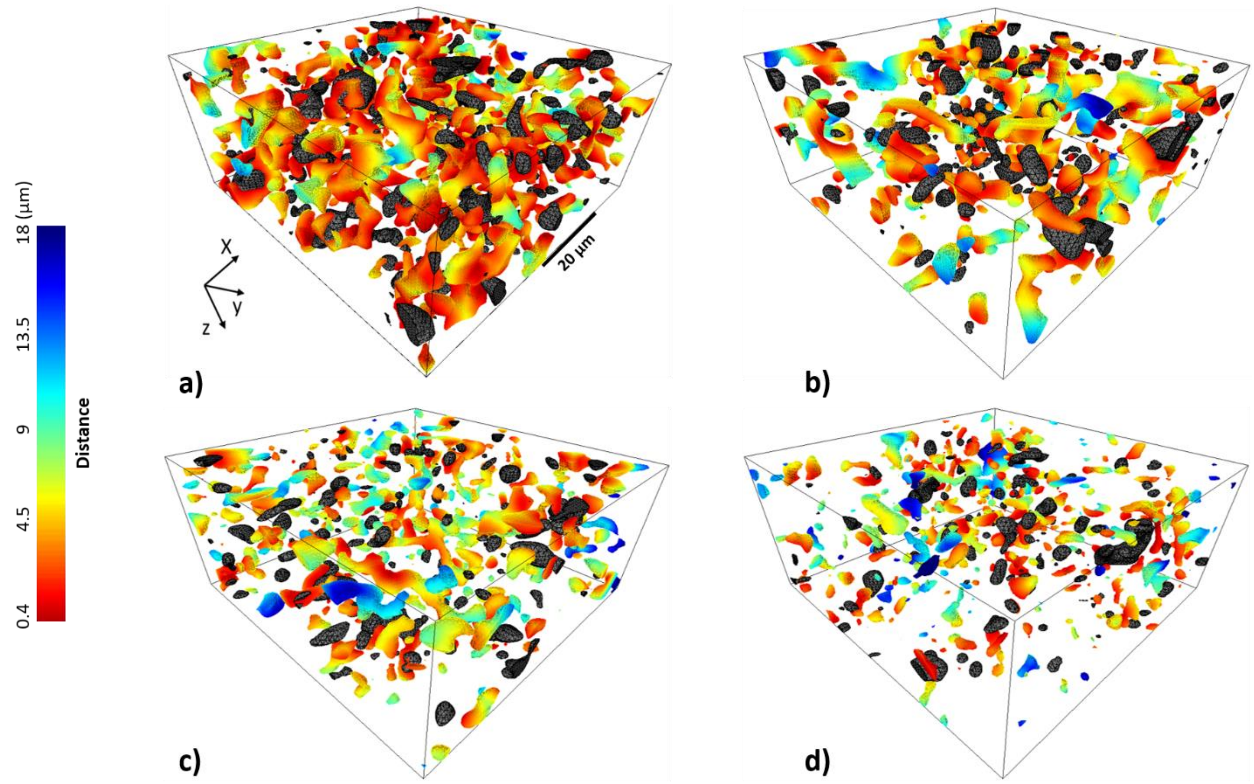

Supplementary Figure 3| Hausdorff distance (d_H) between pore network and a-Si/c-$FeSi_2$ particles. 3D visualization of d_H as well as a-Si/c-$SiFe_2$ meshed particles (black) for (a) pristine, (b) anode with 3 cycles (c) 100 cycles, and (d) 300 cycles, respectively. Red indicates close distance of pore to the next a-Si/c-$FeSi_2$ compound particles while blue shows far distance. The minimum, maximum and mean norm distance changes from 0.49 µm, 12.73 µm, and 3.62 µm to 0.67 µm, 17.53 µm, and 5.37 µm for the pristine anode and anode with 300 cycles, respectively. Scale bar indicated in a) is also valid for c) - d).

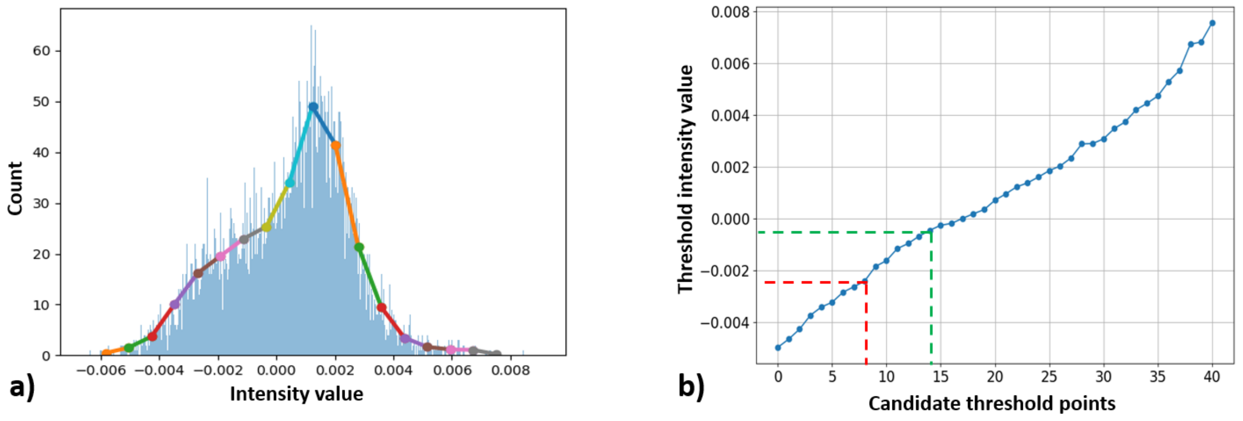

Supplementary Figure 4| The developed histogram-based global threshold method.

(a) Histogram for the slice image with split up intervals illustrating the intensity values in different colors, (b) local extrema points from each interval which define possible candidate threshold points. The candidate threshold represented by the red dotted line is used to segment the pore phase. The candidate threshold represented by the green dotted line is used for over segmenting of the pore phase.

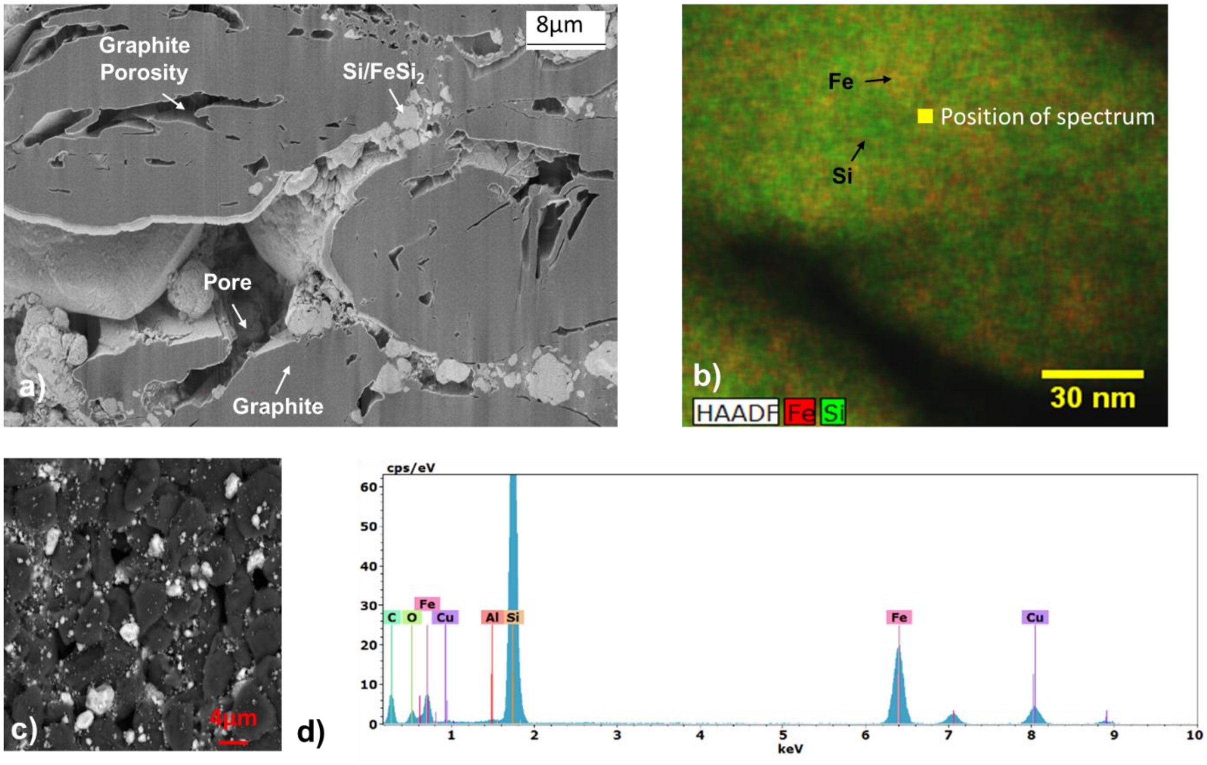

Supplementary Figure 5 | Quantification of the a-Si/c-$FeSi_2$ anode using FIB-SEM combined with STEM-EDX. (a) FIB-SEM slice view from Secondary Electron-detector, showing a representative slice image from the pristine electrode with its main components labeled in the image. (b) STEM-EDX chemical mapping of the a-Si/c-$FeSi_2$ particles with Si and Fe labeled as green and red respectively. (d) EDX spectra of the marked region confirming the presence of Si and Fe peaks. For the chemical mapping of the negative anode we use an FEI-Osiris Scanning transmission electron microscopy (STEM) microscope operated at 200 kV and equipped with a Brücker energy dispersive x-ray (EDX) system consisting of four silicon drift detectors, which ensures a high signal-to-noise ratio. The chemical mapping of the various phases was obtained by generating EDX maps with a lateral resolution of ~30 nm. (c) SEM image of the surface of an anode with 500 cycles, still showing pore sizes with diameters of several μm.

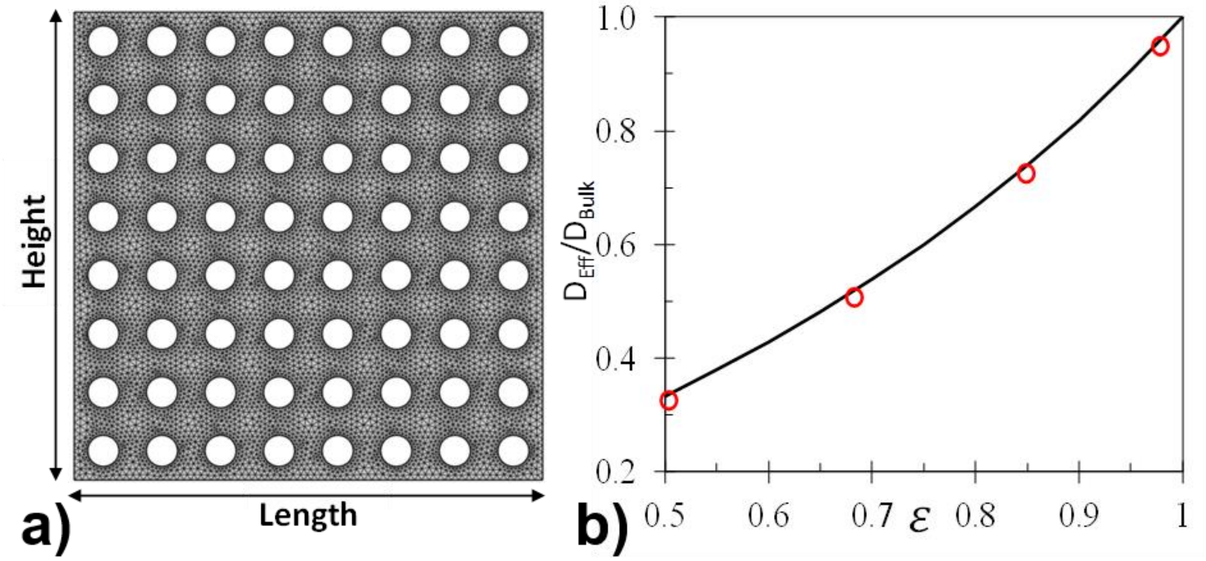

Supplementary Figure 6| Two-dimensional simulation domain (a) used for validating continuum simulations. Comparison of normalized effective diffusion coefficient for continuum simulation (solid line) with appropriate analytical bound (red markers) as a function of accessible volume fraction (ε).

SUPPLEMENTARY NOTES

Supplementary Note 1: Electrode preparation

For an optimized and homogenous paste it is necessary to disperse the conducting agent into the binder solution (hereinafter referred to as "*C-paste*").

Preparation of the *C-paste*:

(i) The conducting agent and the binder (predissolved, 8.0wt% in water) are mixed in a dissolver mixer at 1200rpm for 30min.

Mixing Procedure:

(i) 2/3 of the graphite and the whole amount of the silicon based active material are put in a Hivis Mix Modell 2P-03/1 and is mixed with half of the *C-paste* for 30min at 50rpm.

(ii) In the next step the rest of the graphite and *C-Paste* are added and mixed for 60min at 60rpm.

(iii) Finally water is added to optimize the viscosity of the electrode paste for further processing and the electrode paste is mixed for 30min at 30rpm.

Afterwards the electrode paste is coated on a *Coatema Smartcoater* with following coating parameters:

Cu-foil feed rate	0.5m/min
doctor blade gap	160µm
Drying zone temperatures [°C/°C/°C]:	80/90/110

Supplementary Note 2: FIB-SEM data processing and Segmentation

We use Avizo® to remove curtaining artefacts with the help of FFT and an in-house developed Python script to perform the segmentation. After the pre-processing of the image data, the 3D image are converted to float and normalized between 0 and 1. Next, we use Felzenszwalb[1] algorithm to separate the slice images into regions. This graph-based algorithm tries to find the borders of objects by looking at local differences as well as global (for each slice) differences to decide where to draw a boundary line. For each region, we calculate different features (*i.e.* mean grey value, standard deviation, number of pixels above/below a certain threshold, etc.) and sequentially narrow down the criteria for each feature to fit our system (*e.g.* a region is considered graphite if mean grey value is between 0.4 and 0.6 and the std is below a threshold and so on). When we found good conditions for a specific phase (background, graphite, (SEI/C/B), a-Si/c-$FeSi_2$) we assign the corresponding region a label (0,1,2 or 3). After each assignment we iterate over the remaining regions to further narrow down the conditions. We choose to use this iterative approach to avoid mislabeling of shine-through artefacts as best as possible. At the end, we obtain a mask image for the different phases (pores, graphite, SEI and Si/$FeSi_2$ compound).

Supplementary Note 3: Image Analysis and Segmentation of µ-Synchrotron Data

The measured synchrotron data was performed with an effective voxel size of 0.65 x 0.65 x 0.65 μm^3 and shows scattering artefacts in the vicinity of the copper current collector; to avoid those artefact affected areas in the segmentation, we mask the Cu substrate as follows: Firstly, we apply the median filter to the data. Then a threshold for the copper current collector is applied using the filtered data. Next, the morphological operation dilation, is utilized to get a mask from the binarized substrate. Finally, the original data is masked by replacing the intensity values of the obtained subtracted mask with the background value.

For further analyses, we select representative VOIs (31.85 x 74.75 x 74.75 μm^3) from each 3D reconstructed dataset. Each 3D image sample shows a rather homogeneous morphology. We develop a segmentation method based on a histogram based global iterative threshold multistage approach to find objectively the grey value threshold. The following steps are carried out to segment the four phases (pore-, Si/$FeSi_2$-, intermediate-, and graphite-domain) as shown in Figure (5a and b):.

Pore-domain: A histogram-based global thresholding method is applied to each VOI to segment the pore structure (see Supplementary Figure 4). To binarize the volume, we apply the lowest threshold which is chosen from the drastically slope changes of the relative extrema, calculated from a pre-defined subdivisions of the histogram for each VOIs slice image (red dotted line in Supplementary Figure 4). 3D labelling is applied to the obtained binarized volume to determine the pore-phase.

Si/FeSi$_2$-domain: We separate the Si/FeSi$_2$-domain using a similar procedure as for the pore-phase. However, for the Si/FeSi$_2$-domain the highest threshold is chosen.

Intermediate-domain: To determine the observed continuous domain which surrounds the Si/FeSi$_2$- and pore--domain with highest possible accuracy, we apply two different thresholds to over-segment the pores and Si/FeSi$_2$ together with their surrounded binder and carbon domains (CBDs). Subsequently, we subtract the pore- domain and Si/FeSi$_2$-domain labeled images from their related over-segmented labeled images. The summation of the obtained subtractions (pores and Si/FeSi$_2$) provides the so-called intermediate-domain. It is associated with the CBD as well as domains smaller than the resolution limit (0.65 μm) of the μ-SCT for the pristine anode. For the cycled anode we argue, due to the significant increase of the intermediate domain, that the domain is extended by the emerging SEI. Pore and a-Si/c-FeSi$_2$ domains are decreasing with cycling.

Graphite-domain: This domain was obtained by subtracting the pore-, and Si-phase from intermediate- domain in the representative VOI's. Vol% of graphite domain does not change with cycling.

Adding up the four phases provide a total of 100 Vol%. An iterative validation of the segmentation is done via the comparison of the segmented data and a sufficient statistical amount of grey value slice images.

Supplementary Note 4: Simulation method

The mathematical model is created to capture the influence of silicon and graphite particles, carbon binders, and SEI layer volume fractions on the li-ion diffusion rates. Since we are mainly interested in how the proximate morphology of the silicon particles, and domain micro porosity would affect the Li-ion diffusion, we need not to take into account the full electrochemical equation setup[2] for battery modelling. Hence, our modelling approach is based on a time-dependent classical Fick's 2nd law diffusion equation that governs the lithium-ion concentration $c_{Li}(x,t)$ on a domain Ω_m. For simplicity, we ignore the effect of electric migration on the diffusion coefficient and diffusion in the active material particles,

$$\varepsilon \frac{dc_{Li}}{dt} = -\nabla \cdot D_{Li} \nabla c_{Li} \quad on\ \Omega_m\ , \qquad (1)$$

where D_{Li} is the Li-ion diffusion coefficient and ε is the porosity value of the domain to reflect the influence of the porosity (area fraction) acting on the Li-ion diffusion.

The domain porosity value (for the SEI/C/B), for the corresponding SEM image (main text Figure (7)) configuration, is calculated numerically in COMSOL as 0.63985. This is based on eq. (2) where H and L are the height and length of the domain (5 x 8 μm²) and $f(x,y)dxdy$ represents the elemental area of the SEI/C/B phase.

$$\varepsilon = \frac{1}{L \times H} \iint_{0\,0}^{L\,H} f(x,y)dxdy \qquad (2)$$

To solve eq. (1), appropriate boundary conditions are required. On the rigid surfaces of silicon/graphite particles the normal ionic flux for Li-ion is zero, $\mathbf{n} \cdot \boldsymbol{N_{Li}} = 0$, where $\mathbf{n}$ and $\boldsymbol{N_{Li}}$ are the unit outer normal vector and total flux, respectively. On the outer boundary on the rectangular domain, we use $-\mathbf{n} \cdot \boldsymbol{N_{Li}} = k_f(C_{Li} - c_{Li})$, where k_f, C_{Li} and c_{Li} are, respectively,

mass transfer coefficient, concentration in a bulk solution outside of the porous structure and instant Li-ion concentration. We assume that the Li-ion concentration maintains its bulk concentration at the pores, $c_{Li} = C_{Li}$.

For the validation of the simulation methodology, we demonstrate the accuracy of our model for an inert diffuser in a two-dimensional domain with obstacles, see Supplementary Figure 6a. The effective diffusion coefficient is approximated using analytical bounds and is also explicitly solved using the time-dependent diffusion eq.1 in the presence of obstacles to calculate the effective diffusion coefficient. Supplementary Figure 6b shows the normalized effective diffusion constant of an inert diffuser predicted via Maxwell-Garnett (MG) (eq.3) analytical bound for cylindrical particles in two-dimensional cylindrical obstacles with accessible volume fractions (porosities) ranging from $\varepsilon = 0.5$ to 1.0. For $\varepsilon \rightarrow 1.0$, D_{eff} approaches the bulk rate. We find comparable D_{eff} predictions based on our explicit time-dependent simulations of the continuum diffusion equation.

$$D_{MG} = \frac{\varepsilon}{(2-\varepsilon)} \qquad (3)$$

Supplementary Note 5: elaboration on diminishing behavior for the SEI growth

The SEI growth is bound to the available surface area and the “open” Si/C. At the beginning, there is a lot of open surface area and Si/C. Therefore, the initial evolution of the SEI is quite large, but after each cycle, the amount of surface area and Si/C available for new SEI formation decreases until it reaches a limit, which will lead to the growth becoming zero. By that time, the

Li-ion transport is already not properly functioning anymore. From this, we assume that the growth of the SEI follows a logistic behavior.

References

1. Felzenszwalb, P. F. & Huttenlocher, D. P. Seg-Ijcv. 1–26 (2015). doi:10.1023/B:VISI.0000022288.19776.77
2. Fuller, T. F., Doyle, M. & Newman, J. Simulation and optimization of the dual lithium ion insertion cell. *J. Electrochem. Soc.* **141**, 1–10 (1994).

Corresponding author

Correspondence to Roland.Brunner@mcl.at.
